# Therapeutic Phytogenic Compounds for Obesity and Diabetes

**DOI:** 10.3390/ijms151121505

**Published:** 2014-11-21

**Authors:** Hee Soong Jung, Yun Lim, Eun-Kyoung Kim

**Affiliations:** 1 Department of Brain Science, Daegu Gyeongbuk Institute of Science & Technology, 333, Techno Jungang-daero, Hyeonpung-myeon, Dalseong-gun, Daegu 711-873, Korea; E-Mails: harryjung@dgist.ac.kr (H.S.J.); limyun@dgist.ac.kr (Y.L.); 2 Neurometabolomics Research Center, Daegu Gyeongbuk Institute of Science & Technology, 333, Techno Jungang-daero, Hyeonpung-myeon, Dalseong-gun, Daegu 711-873, Korea

**Keywords:** phytogenic compounds, obesity, diabetes

## Abstract

Natural compounds have been used to develop drugs for many decades. Vast diversities and minimum side effects make natural compounds a good source for drug development. However, the composition and concentrations of natural compounds can vary. Despite this inconsistency, half of the Food and Drug Administration (FDA)-approved pharmaceuticals are natural compounds or their derivatives. Therefore, it is essential to continuously investigate natural compounds as sources of new pharmaceuticals. This review provides comprehensive information and analysis on natural compounds from plants (phytogenic compounds) that may serve as anti-obesity and/or anti-diabetes therapeutics. Our growing understanding and further exploration of the mechanisms of action of the phytogenic compounds may afford opportunities for development of therapeutic interventions in metabolic diseases.

## 1. Introduction

### 1.1. History of Natural Compounds

Living organisms found in nature have chemical compounds or substances collectively termed natural compounds [1]. The various sources of natural compounds include plants, animals, and microorganisms [2]. In this review, we deal with natural compounds in general but mainly focus on plant natural compounds, otherwise known as phytogenic compounds. Phytogenic compounds have been used for treatment of many diseases for millennia. Aspirin was first extracted from the willow tree by Hippocrates in the fifth century BC; its uses include relief of fever and pain, and in childbirth [3]. Morphine from poppies was isolated in 1806, and is useful in the treatment of pain [4]. Natural compounds are usually crude extracts featuring a mixture of either fresh or dried material. Various solvents and several hundred different ingredients can be present. While traditional extraction techniques were too crude and slow to purify each single compound [5], advancements in basic science and technology, such as nuclear magnetic resonance, high-performance liquid chromatography, and mass spectrometry, has made analysis of natural compounds more efficient [6,7].

### 1.2. Advantages of Natural Compounds

Natural compounds have been a good source of new pharmaceuticals for a long time. Approximately 50% of the drugs approved by the Food and Drug Administration (FDA) are phytogenic compounds or derivations. Natural compounds have been crucial in drug development [8,9]. Morphine, vinblastine, vincristine, quinine, artemisinin, etoposide, teniposide, paclitaxel and camptothecin are examples of pharmaceuticals derived from natural compounds [8]. Natural compounds have been a good source for developing new pharmaceuticals because of their vast diversity. This characteristic of natural compounds enables the synthesis of drugs that differ from other chemical compounds in terms of their complex structures and biological potency [2,10]. Additionally, natural compounds are used for drug development and to identify and study targets and pathways involved in disease [2].

### 1.3. Disadvantages of Natural Compounds

Pharmaceutical companies use high throughput screening (HTS) for drug development [2,8]. Enzyme or receptor-based assays are used to simultaneously screen thousands of compounds to uncover possible candidates. However, these methods pose a problem for natural compounds because crude extracts contain hundreds of compounds. Among them, tannins are particularly problematic as they can bind non-specifically to proteins, producing false positive results. For this reason, some natural compounds cannot be used in HTS, unless they are detanninized [8].

Isolating bioactive compounds from raw sources is another problem when dealing with phytogenic compounds [2,8]. Natural compounds in general are available in small quantities [2]. Preclinical development can require quantities from several grams to hundreds of grams. Kilograms of raw material are most likely needed for clinical use. The quantity of raw material required for lead compound extraction varies depending on the potency of the compound and its target [8]. This characteristic of natural compounds delays the development of pharmaceuticals [2]. However, this hurdle can be overcome with synthetic chemistry [8]. Plant-derived anti-cancer drugs including paclitaxel, eribulin, and trabectedin are synthesized using other non-natural sources. For example, paclitaxel was usually isolated from the bark of Pacific yew in the past. Now, but it is produced by plant tissue culture [11].

## 2. Obesity and Diabetes

Despite different symptoms, obesity and diabetes have some similarities. A large percentage of obese patients have insulin resistance [12]. Insulin resistance is a key feature of diabetes. Many, but not all, diabetic patients are obese or have increased abdominal fat. Due to these similarities, it is anticipated that pharmaceutical treatments that simultaneously address obesity and diabetes can be developed. Many studies have revealed natural compounds that affect obesity or diabetes. Only a few have involved natural compounds. The objective of this study is to compile information and analyze natural compounds with potential for development into treatments for obesity and diabetes.

### 2.1. Obesity: Current Issues and Treatments

Obesity is an increasing global problem, especially in developed countries. The World Health Organization (WHO) defines an obese individual as someone whose body mass index (BMI) is over 30 kg/m^2^ [13]. Under 25 kg/m^2^ is considered as normal and between 25 and 30 kg/m^2^ is categorized as overweight. Obesity can lead to various chronic diseases, such as hypertension, type 2 diabetes (T2DM), hyperlipidemia, and coronary artery disease.

The causes of obesity are diverse but generally there are four main factors: the amount of food/drink intake, physical activity (exercise), genetic factors [14], and medical problems [15]. Food/drink intake involves caloric input and physical activity involves caloric output. A healthy combination of these factors produces caloric balance. Sedentary lifestyle and copious intake of food as well as eating unhealthy food disturbs the caloric balance and brings about obesity. Metabolism is also a key factor of obesity. In general, the metabolic rate is lower in obese people than those who are lean. The low metabolic rate does not change when obese people lose weight [16]. For that reason, formerly obese people must be vigilant about their food intake because they can easily regain weight. Genetic alteration is the cause of obesity in a relatively small segment of obese individuals. Obesity can be caused when genes related to leptin, leptin receptor, and melanocortin systems are mutated [13]. Leptin is a hormone synthesized by adipose tissue. This hormone controls food intake and energy expenditure in the hypothalamus of the brain. The expression level of the *ob* gene, which encodes leptin, is dependent on the amount of body fat. When mutation occurs in this gene, leptin regulation is disturbed. The *FTO* (fat mass and obesity associated) gene, whose function remains unknown, can contribute to a 3–5 kg weight difference in obese individuals according to its DNA-sequence variations [16]. Also, *FTO* variation is related to BMI and implies level of obesity from childhood to adulthood [17]. People with diseases such as Down syndrome show a higher prevalence of obesity than others [15]. In this case, obesity is reported as a phenotype. In addition, patients with Cushing’s syndrome show a high frequency of truncal obesity [18] and thyroid abnormal functions like hypothyroidism relate to morbid obesity [19]; however, whether obesity precedes disease or vice versa is unclear.

Several methods are used to treat obesity. One approach is to suppress appetite to decrease food intake [20]. Many factors including neuronal and hormonal regulation contribute to appetite control. Central and peripheral peptides and hormones are involved in controlling food intake [21]. In general, food intake is increased when an orexigenic signal is activated such as Neuropeptide Y (NPY), Agouti-related peptide (AgRP), orexin, and ghrelin. In contrast, when an anorexigenic signal is activated, food intake decreases. Melanocyte-stimulating hormone (α-MSH), insulin, leptin, peptide YY3-36, obestatin, cholecystokinin (CCK), glucagon-like peptide (GLP), and serotonin are typical anorexigenic hormones and peptides. Many pharmaceutical firms have explored their prowess in treating obesity through exploiting the mechanism of these hormones and neuronal signals. FDA-approved medications for obesity include the gastric and pancreatic lipase inhibitor orlistat (Xenical), the endocannabinoid receptor blocker rimonabant (Acomplia), and the monoamine-reuptake inhibitor sibutramine (Reductil) [22]. The endocannabinoid system has two types of receptors: CB1 and CB2. Rimonabant was the first CB1 receptor blocker discovered [23]. The brain endocannabinoid system is related to a reward mechanism and involves regulating the orexigenic and anorexigenic pathways [24]. Sibutramine inhibits the reuptake of serotonin and noradrenaline [25]. Orlistat remains in use. Rimonabant and sibutramine have been withdrawn and are not available in most countries [25] because of dangerous side effects such as serious psychiatric disorders, heart attack, and stroke. A side effect of orlistat is fatty oily stool. General side effects of anti-obesity drugs include constipation, dry mouth, and insomnia. Finding a new drug with no side effects is the ultimate goal for many pharmaceutical companies. Natural compounds are renowned for their fewer side effects compared to synthetic pharmaceuticals [1]. For this reason, natural compounds could be good candidates for developing obesity treatments. Natural bioactive substances are natural compounds that can affect biological processes or substrates. Hence, natural compounds have an impact on function of the human body and ultimately human health [26]. Among all the natural compounds, this review will concentrate on phytogenic compounds.

### 2.2. Diabetes: Current Issues and Treatments

The hallmark of diabetes is hyperglycemia caused by a defect in insulin secretion and/or insulin action [27]. Chronic hyperglycemia can lead to cardiovascular disease, retinopathy, neuropathy, nephropathy, and diabetic foot disease [4]. The cause of diabetes can be varied. For example, autoimmunity can destroy pancreatic β-cells, which induces insulin deficiency. Abnormality in insulin recognition can lead to insulin resistance. This circumstance impairs signal transduction in insulin signaling, which will eventually result in hyperglycemia. Both physiological defects are found in many diabetic patients. However, it remains unknown which is the key cause of hyperglycemia [27].

Diabetes can be classified into various groups, but most common are type 1 and 2. Type 1 diabetes (T1DM) is caused by insufficient insulin secretion due to autoimmune destruction of β-cells. Only 5%–10% of diabetic patients have T1DM. T2DM is a consequence of insulin resistance and lack of appropriate response to hyperglycemia. Most (90%–95%) of diabetic patients have T2DM. Some diabetic patients are able to manage their blood glucose level with exercise, proper diet, and oral glucose-lowering agent depending on their condition. These diabetic patients do not require external insulin to survive. On the other hand, those with severe β-cell damage cannot manage their blood glucose level with simple exercise and diet. They need regular injections of insulin to survive [27].

Most T2DM patients do not need exogenous insulin to manage their blood glucose level. This constitutes non-insulin dependent diabetes (otherwise known as adult-onset diabetes). Many T2DM patients are obese. Many of those who do not need exogenous insulin have increased body fat in the abdomen. Often it is undiagnosed for many years because hyperglycemia advances slowly and it is difficult for the patient to notice the symptoms of diabetes [27].

Treatment for T2DM is continuously being developed. The most common pharmaceutical prescribed for T2DM is metformin [28,29]. Metformin reduces hepatic glucose output and plasma insulin level, and enhances insulin sensitivity of periphery tissues, thereby increasing glucose uptake [29]. Metformin is a hypoglycemic agent that reduces the mortality rate of T2DM patients. Side effects of metformin are nausea, diarrhea, and flatulence [28]. Other drugs have been developed for diabetes treatment, but none is as effective and/or as potent as metformin [28]. Lifestyle intervention is another treatment for diabetes. In some cases, lifestyle intervention is more beneficial than pharmaceuticals. Lifestyle intervention consists of low calorie and low fat diet, and a minimum of 150 min of moderately intense physical exercise per week [30]. This may suggest that phytogenic compounds could be better suited for diabetes treatment than chemically synthesized pharmaceuticals.

## 3. Phytogenic Compounds

**Figure 1:**
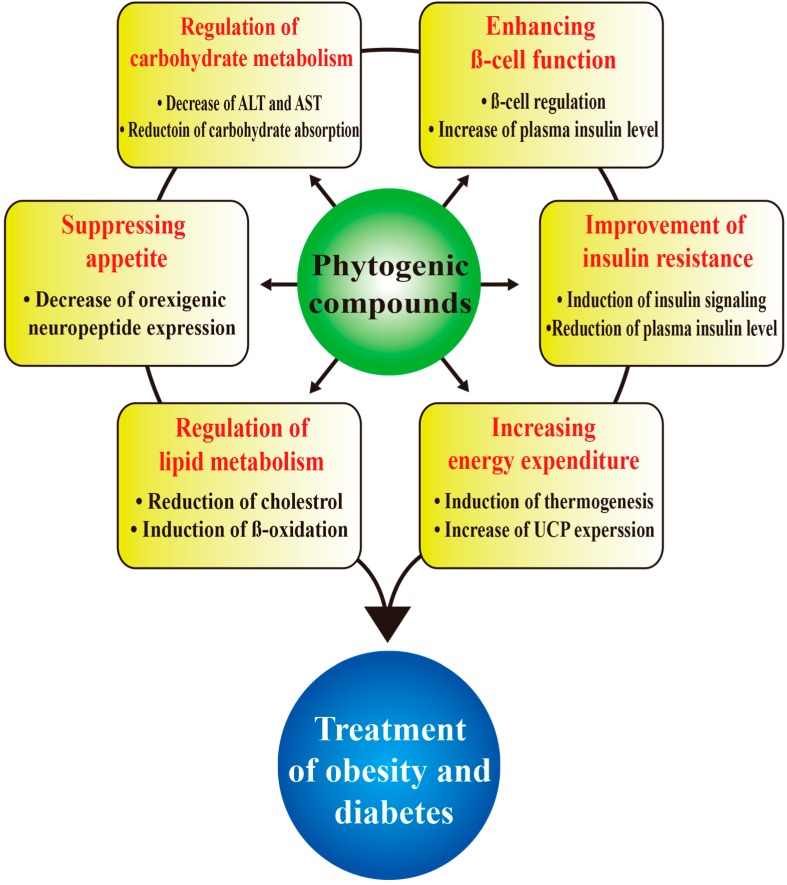
Effects of phytogenic compounds on diabetes and obesity. Possible outcome of phytogenic compounds as potential candidates for development of obesity and diabetes treatments are illustrated.

Phytogenic compounds have been used to treat various diseases for decades [8,9]. Thousands of phytogenic compounds have been studied for obesity and diabetes treatment. The phytogenic compounds discussed in this review are potentially valuable in the treatment of obesity and/or diabetes. Various effects of phytogenic compounds are discussed below and also illustrated in Figure 1.

### 3.1. Possible Therapeutic Compounds for Obesity

#### 3.1.1. Compounds that Suppress Food Intake

##### *Panax quinquefolium* (American Ginseng)

Ginsenoside is an anti-obesity compound extracted from ginseng. *Panax* *ginseng* (Asian ginseng) and *Panax quinquefolium* (American ginseng) contain many ginsenosides. Ginsenoside Rb1 has many therapeutic actions on the glucose metabolism and lipid metabolism. When ginsenoside Rb1 (10 mg/kg) was intraperitoneally injected to normal diet fed mice and high fat diet (HFD) fed mice groups for 3 weeks, it reduced body weight, total food intake, fat contents, serum leptin, and serum nitric oxide equal to or lower than the normal diet group [31]. Also, the paraventricular nucleus in the hypothalamus showed decreased expression of orexigenic NPY and increased anorexigenic CCK when ginsenoside Rb1 was treated to the HFD group. There are many types of ginsenosides in *P. ginseng* and they also have a role in inhibiting pancreatic lipase (Table 1). This will be further discussed in Section 3.1.3.

##### *Panax ginseng* (Asian Ginseng)

The concentrations of ginsenoside Re, Rb2, and Rd in *P. ginseng* are significantly higher in berry than root [32]. Among them, ginsenoside Re increases energy expenditure by regulating thermogenesis [33]. Extract of ginseng berry (150 mg/kg) was intraperitoneally injected to obese C57BL/6J *ob*/*ob* mice for 12 days. Ginsenoside Re-treated *ob*/*ob* mice reduced food intake (15%) and body weight (11.6%) significantly. In addition, body temperature (2.8%) and energy expenditure (35%) increased significantly in *ob*/*ob* mice [33].

**Table 1:** Phytogenic compounds with potential for the development of treatments for obesity and diabetes

Scientific Name (Common Name)	Methods	Results	Function							Ref.
			Diabetes			Obesity				
			Improve Insulin Resistance	Enhance β-Cell Function	Multiple Anti-Diabetic	Suppress Appetite	Stimulate Energy Expenditure	Regulate Lipid Metabolism	Regulate Carbohydrate Metabolism	
*Vaccinium* spp. **(Blueberry)**	Blueberry powder fed to HFD-induced obese mice	↓Blood glucose level	●	-	-	-	-	-	-	[26]
	Oral administration of blueberry with Labrasol	↓Blood glucose level	●	-	-	-	-	-	-	[40]
	Fermented blueberry fed to KKA^y^ mice	↓Blood glucose level	●	-	-	-	-	-	-	[41]
	Blueberry fed to Zucker rats	↑PPAR-α and PPAR-γ activity	●	-	-	-	-	-	-	[42]
	Oral administration of blueberry extract to rats	↓Food intake and body weight gain	-	-	-	●	-	-	-	[43]
	Oral administration of water containing with blueberry extract to HFD fed mice	↓Total body fat and body fat	-	-	-	●	-	-	-	[44]
*Vaccinium angustifolium* **(Wild blueberry)**	Wild blueberry-enriched diet for obese rats	↓Triacylglycerol, total cholesterol, SREBP-1 and fatty acid synthase ↑PPAR-α and PPAR-γ	-	-	-	-	-	●	-	[45]
*Vitis vinifera* **(Grape vine)**	Resveratrol treated to C2C12 mytotube cell line.	↑Glucose uptake and AMPK	-	-	●	-	-	-	-	[46]
	Polyphenolic extract treated to HepG2 cell line	↓Glycogen phosphorylase	-	-	●	-	-	-	-	[47]
	Resveratrol treated to L6 rat skeletal muscle cell line	↑Glucose uptake and AMPK	-	-	●	-	-	-	-	[48]
*Cinnamomum* **(Cinnamon)**	Cinnamon treated to rat adipocytes	↑PI3K	●	-	-	-	-	-	-	[49]
	Water and polyphenol extracts treated to 3T3-L1 adipocytes	↑Insulin receptor and GLUT4 protein expression	●	-	-	-	-	-	-	[50]
	Administration of cinnamon extract to 3T3-L1 adipocytes	↑The expression of LPL, CD36, GLUT4, and acyl-CoA oxidase	-	-	-	-	-	●	-	[51]
	Administration of cinnamon powder with water to C57BL/6J *db/db* mice	↓Fasting glucose level, free fatty acid, LDL cholesterol, and AST levels	-	-	-	-	-	●	-	[51]
*Trigonella foenum graecum* **(Fenugreek)**	Oral administration of fenugreek seed to diabetic patients	↓Blood glucose level ↑Plasma insulin level	●	-	-	-	-	-	-	[52]
	Oral administration of fenugreek to NIDD patients	↓Fasting blood glucose level	●	-	-	-	-	-	-	[53]
*Ervatamia microphylla* **(Kerr)**	Oral administration of conophylline to STZ-induced diabetic rats	↓Blood glucose level ↑β-cell proliferation	-	●	-	-	-	-	-	[54]
	Conophylline treatment to pancreatic stellate cells	↓Activation of pancreatic stellate cells	-	●	-	-	-	-	-	[55]
	Conophylline administration to Goto-Kakazaki rats	↓Blood glucose level ↑Plasma insulin level	-	●	-	-	-	-	-	[55]
*Anoectochilus roxburghii* **(Jewel orchid)**	Oral administration of kinsenoside to STZ-induced rats	↓Blood glucose level ↑Plasma insulin level	-	●	-	-	-	-	-	[56]
*Carica papaya* **(Papaya)**	Papaya extract administered to STZ-induced mice	↓Blood glucose level ↑Insulin synthesis and β-cell regeneration	-	●	-	-	-	-	-	[57]
*Momordica charantia* **(Bitter melon)**	Intraperitoneal injection of bitter melon aqueous to STZ-induced mice	↓Blood glucose level	-	-	●	-	-	-	-	[58]
	Oral administration of bitter melon juice to maturity onset diabetes patients	↓Blood glucose level	-	-	●	-	-	-	-	[59]
	Oral administration of bitter melon powder to HFD fed mice	↓Body weight gain, hyperlipidemia, and hyperglycemia	-	-	-	-	-	●	-	[60]
	Oral administration of bitter melon powder to HFD-induced obese rats	↓The number of large adipocytes, adipose tissue mass, TAG content, FAS, ACC-1, LPL and adipocyte fatty acid-binding protein (aP2)	-	-	-	-	-	●	-	[61]
*Capsicum* **(Chili pepper)**	Capsaicin administered to ZFD rats	↓Blood glucose level and Plasma insulin level	-	-	●	-	-	-	-	[62]
	Oral administration of chili pepper powder to HFD-induced diabetic rats	↑Plasma insulin level	-	-	●	-	-	-	-	[63]
	Administration of capsaicin to 3T3-L1 adipocytes	↑Hormone sensitive lipase, CPT-1a and UCP2	-	-	-	-	●	-	-	[64]
	Oral administration of capsinoids to men and give a cold exposure to them	↑BAT activity	-	-	-	-	●	-	-	[65]
	Calorie restricted diet with capsaicin or without capsaicin fed to human	↑Resting energy expenditure and diet-induced thermogenesis (capsaicin containing group)	-	-	-	-	●	-	-	[66]
*Glycyrrhiza* **(Liquorice)**	Synthetic amorfrutins administered to HFD-induced obese mice	↓Blood glucose level, Plasma insulin level and body weight	●	-	-	-	-	-	-	[67]
	Oral administration of ethanol extract to KKA^y^ mice	↓Blood glucose, weight and intra-abdominal adipose tissue	●	-	-	-	-	-	-	[68]
	Oral administration of licorice flavonoid oil to HFD-induced obese mice	↓Genes related to acetyl-CoA synthesis and lipid biosynthesis, weight of abdominal white adipose tissues, and body weight gain ↑Genes related to β-oxidation and acetyl-CoA degradation.	-	-	-	-	-	●	-	[69]
*Dioscoreaceae* **(Dioscorea)**	Dioscorea polysaccharide treated to TNF-α-induced insulin resistant mouse liver cell line	↑Glucose uptake and activate insulin signaling	●	-	-	-	-	-	-	[70]
	Oral administration of dioscorea extract to fructose-induced insulin resistant Wistar rats	↓Blood glucose level and Plasma insulin level	●	-	-	-	-	-	-	[71]
*Nymphaea stellata* **(Egyptian lotus)**	Oral administration of lotus extract to STZ-induced diabetic rats	↓Blood glucose ↑Plasma insulin level and number of β-cell mass	-	●	-	-	-	-	-	[72]
*Nelumbo nucifera* **(Indian lotus)**	Oral administration of lotus leaves extract to HFD-induced obesity mice and rats	↓Pancreatic lipase, α-amylase, α-glucosidase, total cholesterol, triglycerides, and LDL cholesterol ↑HDL cholesterol	-	-	-	-	-	●	-	[73]
	Oral administration of lotus seeds extract to HFD-induced obesity mice	↓α-amylase, α-lipase, body weight gain and triglycerol ↑Expression of UCP3 mRNA in C2C12 myotubes	-	-	-	-	●	-	-	[74]
*Silybum marianum* **(Milk thistle)**	Oral administration of silymarin to alloxan-induced diabetic rats	↓Blood glucose, ↑Pancreatic SOD, GSHPx and CAT	-	●	-	-	-	-	-	[75]
	Oral administration of silymarin seed extract to T2DM patients	↓Fasting blood glucose and glycosylated hemoglobin level.	-	●	-	-	-	-	-	[76]
	Oral administration of silymarin as adjuncts to glibenclamide	↓Postprandial and fasting blood glucose level, and glycosylated hemoglobin	-	●	-	-	-	-	-	[77]
*Panax quinquefolium* **(American ginseng)**	Intraperitoneal injection of Rb1 to HFD-induced obese rats	↓Body weight, total food intake, fat contents, serum leptin, serum nitric oxide, and NPY ↑CCK	-	-	-	●	-	-	-	[31]
*Panax ginseng* **(Asian ginseng)**	Intraperitoneal injection of Ginseng berry extract to *ob*/*ob* mice	↓Food intake and body weight ↑Body temperature and energy expenditure	-	-	-	●	●	-	-	[33]
	Intraperitoneal injection of Rb1 to HFD-induced obese rats	↓Food intake, body weight and body fat ↑Energy expenditure	-	-	-	-	●	-	-	[78]
	Intraperitoneal administration of Rb1 to HFD-induced obese rats	↓Liver weight, hepatic triglyceride content, and ACC ↑CPT-1 and AMPK	-	-	-	-	-	●	-	[79]
*Camellia sinensis* **(Green tea)**	Oral administration of EGCG to HFD fed mice	↓Body weight gain, body fat percentage, and visceral fat weight	-	-	-	-	-	●	-	[80]
	Oral administration of EGCG to HFD fed rats	↓Total cholesterol and LDL cholesterol	-	-	-	-	-	●	-	[81]
	EGCG mixed with caffeine, orally administration to human	↓Body weight and body weight gain	-	-	-	-	●	●	-	[82]
*Camellia sinensis* **(Black, green, and mulberry tea)**	Oral administration of the extract of black, green, and mulberry tea to human	↑Breath-hydrogen concentration	-	-	-	-	-	-	●	[83]
*Glycine max Merr* **(Soybeans)**	Oral administration of soybean isoflavone chow to obese rats	↓Plasma glucose, AST, and ALT	-	-	-	-	-	-	●	[84]
*Hoodia gordonii* **(Hoodia)**	Intracerebroventricular injection of the purified P57AS3 to rats	↓Food intake ↑ATP content in hypothalamic neurons	-	-	-	●	●	-	-	[35]
	Oral administration of glycosides 1 and 2 to rats	↓Food intake and body mass	-	-	-	●	-	-	-	[36]
	Oral administration of organic solvent extract to rats	↓NPY ↑CPT-1, T3, and T4	-	-	-	●	●	-	●	[37]

↓: decrease, ↑: increase, ●: having an effect.

##### *Hoodia* *gordonii* (Hoodia)

*Hoodia gordonii* is a thirst quencher [34]; however, its role as an appetite suppressant has only been recently discovered. P57AS3 is an oxypregnane steroidal glycoside extracted from *H. gordonii* and *H. pilifera*. When P57AS3 was injected intracerebroventricularly into the third ventricle, 24 h food intake was reduced by 40%–60% [35]. P57AS3 also increased adenosine triphosphate (ATP) production; therefore there is a possibility that *H. gordonii* could regulate food intake and energy homeostasis. When glycosides 1 and 2 isolated from dried stems of *H. gordonii* were gavaged orally at 6.25–50 mg/kg to rats for 8 days, food intake and body mass were decreased at all doses compared with the control group [36]. Also, *H. gordonii* has effects on diverse factors such as mitochondrial carnitine palmitoyltransferase-1 (CPT-1), thyroid hormones, NPY, and insulin like growth factor-1 (IGF-1) [37]. When male Sprague Dawley rats were orally fed with three different doses (50, 100, 150 mg/kg of body weight) of organic solvent extract from *H. gordonii*, food intake and the level of NPY were decreased dose-dependently. CPT-1 and thyroid hormones such as Tri-iodothyronine (T3) and thyroxine (T4) were increased. CPT-1 is a fatty acid oxidation enzyme that participates in lipid metabolism and the increase of CPT-1 indicates enhanced fatty acid oxidation. Elevation of T3 and T4 indicates an increase in energy expenditure [38] and maintaining glucose homeostasis as their role in promoting carbohydrate metabolism [39]. Thus, *H. gordonii* may participate in suppressing appetite, increasing energy expenditure and lipid metabolism, and promoting carbohydrate metabolism.

##### *Vaccinium* spp. (Blueberry)

Berries are well known for their antioxidant effect. Blueberry has been suggested to improve cardiovascular defects and to have antioxidant anti-obesity and anti-diabetic effects [40,41,85]. Blueberry mostly consists of anthocyanins with the remaining compounds being derivatives of hydroxycinnamic acid, flavonols, flavan-3-ols, folic acid, vitamin C, and fiber [85]. Blueberry extract from two cultivars, “Centurion” and “Maru”, can suppress food intake by increasing satiety [43]. When rats were gavaged water extract of blueberry for 6 days, food intake and body weight gain decreased significantly. In addition, when C57BL/6 mice on HFD drank water containing blueberry extract, total body fat weight and body fat were decreased compared to the HFD control group [44]. Anthocyanins reduced fasting serum glucose concentration of HFD group to normal level. Berry anthocyanins reportedly have better anti-obesity effect than whole blueberries [44,86].

#### 3.1.2. Compounds that Stimulate Energy Expenditure

Energy expenditure includes thermogenesis, physical activity, and obligatory energy expenditure [87]. There are two types of adipose tissue, white adipose tissue (WAT) and brown adipose tissue (BAT). WAT stores extra energy as triglyceride and BAT produces heat. Researchers are trying to discover ways to spend BAT as energy to produce heat for energy expenditure from body. Uncoupling proteins (UCP1, UCP2 and UCP3) are key players in regulating cellular metabolism and they attenuate production of reactive oxygen [88]. UCP1 catalyzes adaptive thermogenesis in mammalian BAT [89]. UCP2 and UCP3 can be thermogenic only when they are activated by appropriate effectors and usually do not respond to adaptive thermogenesis [88].

##### *Nelumbo nucifera* (Indian lotus)

*Nelumbo nucifera* has been used as treatment for many diseases for millennia. Each part of *N. nucifera* has different therapeutic effects that include relief of fever, inflammatory skin conditions, and bleeding disorders [90]. Extract of leaves affects digestive enzyme activity, lipid metabolism, and thermogenesis. When the extract of *N. nucifera* leaves was treated to HFD-induced obesity mice for five weeks, it decreased activities of α-amylase and lipase, and increased lipid metabolism and expression of UCP3 mRNA in C2C12 myotubes [74]. UCP3 is expressed in BAT and skeletal muscle and up-regulation of UCP3 expression increased thermogenesis [91]. In addition, leaves of *N. nucifera* include 11 types of flavonoids: eudesmane sesquiterpene, 13 kinds of megastigmanes, and eight forms of alkaloids [92]. Among them, flavonoids have hypolipidemic effects and inhibit pancreatic lipase, α-glucosidase, and α-amylase [73]. Like *P. ginseng*, *N. nucifera* is involved in lipid metabolism in addition to energy expenditure (Table 1).

##### *Capsicum* (Chili pepper)

Capsaicin is the major compound in *Capsicum annuum*, commonly referred to as red chili peppers. It is widely used as a spice in South East Asia, China, and Latin-America countries [63]. Capsaicin has been reported to increase thermogenesis through increasing secretion of catecholamine from the adrenal medulla [93]. *In vitro*, capsaicin can induce the expression of genes involved in lipid catabolism and thermogenesis, such as hormone sensitive lipase, CPT-1a, and UCP2 [64]. UCP2 uncouples oxidative phosphorylation and increases thermogenesis [93]. The mRNA level of UCP2 increases dose-dependently in response to capsaicin [64]. CH-19 sweet pepper contains capsiate, which has a structure similar to capsaicin but without any pungency [94]. Capsiate increases energy expenditure via activating BAT in humans [65]. Also, capsaicin increases energy expenditure as shown in *in vivo* experiment involving negative balance condition in humans [66]. Subjects were placed in a respiration chamber and provided with a normal diet containing with 2.56 mg of capsaicin or not. Another group was placed under the same conditions except for 25% calorie restriction with the same amount of capsaicin or not. Resting energy expenditure (REE) and diet-induced thermogenesis (DIT) were higher in the calorie restricted capsaicin group than calorie restricted group. Fat oxidation was also increased in the calorie restricted capsaicin group. This result indicates that capsaicin increases REE and DIT even in negative energy balance. Maybe capsaicin can be used to treat obesity for weight loss of people who are on a diet.

#### 3.1.3. Compounds that Regulate Lipid Metabolism

##### *Panax ginseng* (Asian Ginseng)

As mentioned in Section 3.1.1., ginsenoside Rb1 from *P. ginseng* has a role in lipid metabolism. Acute administration of ginsenoside Rb1 reduced food intake, body weight and body fat in HFD-induced obese rats [78]. In this study, Rb1 also increased energy expenditure, decreased hyperglycemia, and enhanced glucose tolerance in HFD-induced obese rats. Also, Rb1 reduced accumulation of liver fat in HFD-induced obese rats [79]. The authors assumed that the reduction was due to increases in fatty acid oxidation and in CPT-1 activity in Rb1-treated hepatocytes. Ginsenoside Rb1 can increase peroxisome proliferator-activated receptor γ coactivator-1α (PGC-1α), peroxisome proliferator activated-receptor (PPAR), CPT-1a, and acyl-CoA oxidase genes that encode enzymes related to lipolysis. Lipogenesis related genes including sterol regulatory element-binding protein 1c (SREBP1c), fatty acid synthase (FAS), acetyl-CoA carboxylase (ACC), and stearoyl-CoA desaturase-1 (SCD-1) were decreased when ginsenoside Rb1 was treated in the study. In addition, ginsenoside Rb1 signaling activated AMP-activated protein kinase (AMPK), inducing ACC phosphorylation for the inhibition of fatty acid synthesis in both cultured primary hepatocytes and in obese rats [79].

##### *Camellia sinensis* (Green Tea)

Epigallocatechin gallate (EGCG) is one of the major catechins in *C. sinensis* [95]. When EGCG was treated (3.2 g/kg diet) to HFD-fed mice for 16 weeks, body weight gain, body fat percentage, and visceral fat weight were reduced compared to only HFD-fed control mice [80]. EGCG treatment also attenuated insulin resistance, plasma cholesterol, liver weight, and liver triglyceride (TG). In addition, short-term treatment of EGCG (3.2 g/kg diet) for 4 weeks decreased mesenteric fat weight and blood glucose comparing with HFD control mice. This result suggests that EGCG is associated with decreasing lipid accumulation in the liver. In another study [81], EGCG lowered total cholesterol (TC) and low density lipoprotein (LDL) cholesterol level in HFD with 1% EGCG fed rats than HFD fed rats. Intestinal cholesterol absorption was decreased in the EGCG group, too. In a meta-analysis study of a clinical trial with EGCG and caffeine [82], EGCG reduced body weight and inhibited body weight gain after weight loss through increasing energy expenditure and fat oxidation when mixed with caffeine. This result showed that ECGC may assist caffeine to increase energy expenditure.

##### *Nelumbo nucifera* (Indian Lotus)

Flavonoids extracted from *N. nucifera* leaves using ethanol can inhibit pancreatic lipase, α-glucosidase, α-amylase, and hypolipidemic effects [73]. In the study, rats and mice were orally treated with *N. nucifera* leaf flavonoids (NLF). NLF decreased TG level in normal rats and decreased the levels of TC and TG in acute hyperlipidemic mice dose-dependently. By inhibiting lipid absorption in the liver, NLF can ameliorate the level of steatosis [73]. Leaves of *N. nucifera* have a strong ability to lower lipid levels. Rhizome of *N. nucifera* is composed of polyphenolic compounds and can alleviate hepatic steatosis in *db*/*db* mice [96]. *N. nucifera* polyphenol from its root suppresses hepatic lipogenesis through decreasing activities of FAS and malic enzyme, which is related to lipogenesis, but not CPT. Seeds of *N. nucifera* contain polyphenolics and flavonoids*. N. nucifera* seed ethanol extract (NSEE) may inhibit the differentiation of human pre-adipocytes into adipocytes [97]. When NSEE was orally administered to HFD fed rats, it suppressed body weight gain and decreased serum TG, leptin, and serum adiponectin levels compared to HFD fed control group. This suggests that NSEE may have an inhibitory effect on adipogenesis. Since the leaf, root, and seed from *N. nucifera* contain flavonoids and polyphenolics, these compounds may have a similar role in suppressing lipogenesis.

##### *Vaccinium angustifolium* (Wild Blueberry)

Wild blueberries contain anthocyanin, which is an antioxidant polyphenol [98]. When a wild blueberry-enriched diet was provided to obese and lean Zucker rats for 8 weeks, the obese Zucker rats showed improvement of dyslipidemia and changes in genes related to lipid metabolism [45]. Triacylglycerol (TAG) and TC levels were significantly decreased in the obese group whereas there was no significant change in the lean group. The expression level of PPAR-α and PPAR-γ, transcription factors of lipid metabolism, increased in abdominal adipose tissue (AAT) of the obese rats. In contrast, SREBP-1 and FAS were decreased in AAT and liver. These findings suggest that wild blueberry improves lipid metabolism by regulating genes related to lipid metabolism.

##### *Cinnamomum* (Cinnamon)

Cinnamon is produced from bark of *Cinnamomum.* Cinnamon extract improves insulin resistance and lipid metabolism by activating of PPAR-α and PPAR-γ [51]. Target genes of PPARs are lipoprotein lipase (LPL), cluster differentiation 36 (CD36), glucose transporter type 4 (GLUT4), and acyl CoA oxidase. Their expression levels were elevated when 3T3-L1 adipocytes were treated with cinnamon extract. When C57BL/6J *db*/*db* mice were gavaged with cinnamon powder dissolved in water, fasting glucose level, free fatty acid, LDL cholesterol, and AST (aspartate aminotransferase) levels decreased because of the activation of PPARs. In *ob*/*ob* mice, cinnamon extract improved insulin sensitivity, insulin-stimulated locomotors activity, and glucose tolerance while insulin secretion was not changed [99]. Water-soluble cinnamon extract can also affect body composition and features of metabolic syndrome [100]. In the latter study, 22 participants with pre-diabetes showed decreased systolic blood pressure, fasting blood glucose, and body fat and increase in lean mass when they took two capsules (250 mg) of Cinnulin PF^®^ twice per day for 12 weeks.

##### *Glycyrrhiza* (Liquorice)

Concerning obesity, licorice flavonoids may suppress the accumulation of abdominal white adipose tissues and body weight gain in HFD-induced obese C57BL/6J mice [69]. When they were fed with HFD containing 2% licorice flavonoid oil (LFO), genes related to β-oxidation and acetyl-CoA degradation were up-regulated more than 2-fold in the liver. Genes related to acetyl-CoA synthesis and lipid biosynthesis were decreased by more than 2-fold. These effects were similar in a rat study [101]. LFO reduced total body fat and visceral fat in overweight people with a BMI of 24–30 [102]. Participants received 300–900 mg of LFO daily for 8 weeks. They displayed significant decreases in body weight, BMI, visceral fat area, and LDL cholesterol.

##### *Momordica charantia* (Bitter Melon)

*Momordica charantia* is a tropical plant commonly found in India, Asia, East Africa, and South America. It is also known as bitter melon [103]. Bitter melon inhibits the development of hyperlipidemia and hyperglycemia. When C57BL/6 mice were fed a HFD supplemented with bitter melon powder for 16 weeks, they showed less body weight gain, hyperlipidemia, and hyperglycemia [60]. Bitter melon treatment recovered the level of mitochondrial dynamics regulators, such as dynamin related protein 1 (DRP1) and mitofusin 1 (MFN1), and pro-apoptotic proteins, such as caspase 3, Bax, and Bcl-xl. When HFD-induced obese rats consumed bitter melon powder, the number of large adipocytes, adipose tissue mass, and TAG contents were decreased compared to the HFD-fed control group [61]. FAS, acetyl-CoA carboxylase-1 (ACC-1), LPL, and adipocyte fatty acid-binding protein (aP2) showed lower levels in the mice fed a HFD supplemented with bitter melon. Bitter melon extract is an agonist of PPAR-α and PPAR-γ [104], and reduces SREBP-1c and resistin [105]. Taken together, the data indicate that bitter melon suppresses lipid accumulation, regulates mitochondrial activity, and inhibits the development of obesity.

#### 3.1.4. Possible Therapeutic Compounds that Regulate Carbohydrate Metabolism

The role of carbohydrate metabolism in the development of obesity is not clearly understood, but many studies show evidence of a relationship between carbohydrate metabolism and obesity. Comparing conventional diet (composed of a high-carbohydrate, low-fat, and low-calorie) group and Atkins diet (composed of low-carbohydrate, high-protein, and high-fat, low-carbohydrate) groups, the Atkins diet group showed a greater weight loss for 6 months in a clinical study [106]. The down-regulation of carbohydrate metabolism, decreased absorption of carbohydrate [83], and inhibition of carbohydrate digestion [107] are potent to ameliorate obesity through increased weight loss and decreased weight gain.

##### *Camellia sinensis* (Teas)

The extract of black, green, and mulberry teas may interrupt carbohydrate absorption by inhibiting α-amylase [108], α-glucosidase [109], sodium-glucose transporters [110,111], and pancreatic lipase [112,113]. In a human study, the extract of black, green, and mulberry teas was orally administrated to healthy volunteers [83]. Breath-hydrogen and ^13^CO_2_ were measured for carbohydrate absorption. Volunteers were orally administrated an extract of black (0.1 g), green (0.1 g), and mulberry (0.1 g) teas in each meal. Participants consumed meals containing lipid and carbohydrate or meals containing only lipid without carbohydrate. Meals containing lipid, carbohydrate and tea extracts increased breath-hydrogen concentration, indicating malabsorption of carbohydrate. On the other hand, no changes were evident in the carbohydrate-free meals group with or without tea extract. This data implies that compounds within tea extract may have an inhibitory effect on carbohydrate absorption.

##### *Glycine max Merr* (Soybean)

Soy has many beneficial effects for obesity and diabetes [114]. Chow supplemented with soybean isoflavones was fed to lean rats and obese SHR/N-cp rats [84]. They were fed 20% casein, 20% casein with 0.1% soybean isoflavone mixture, 20% casein with 0.1% probiotic mixture, and 20% casein with 0.1% isoflavone and 0.1% probiotic mixture. Isoflavones alone decreased plasma glucose, AST, and ALT (alanine transaminase) in both groups. AST and ALT are plasma enzymes related to liver function. They are higher in obese rats than lean rats. High concentration of carbohydrate of the meal increased AST and ALT, than did a high concentration of fat [115]. These studies suggest that soy isoflavones may have an effect on reducing carbohydrate metabolism.

### 3.2. Possible Therapeutic Compounds for Diabetes

#### 3.2.1. Possible Therapeutic Compounds that Regulate Insulin Resistance

##### *Trigonella foenum graecum* (Fenugreek)

*Trigonella foenum graecum* is a leguminous herb widely available in India, Mediterranean, and North Africa. Seeds of fenugreek are used as a spice in India and it is used to make bread with wheat and maize flour. Furthermore, it has been used for medicinal purpose for many years. When fenugreek seeds were administered to diabetic patients, blood glucose level was reduced and plasma insulin level was increased [52]. Another study was conducted with non-insulin dependent diabetes patients [53]. Research candidates were given bread containing equal amounts of ground fenugreek seed for 10 days. Fasting blood glucose and serum insulin levels were reduced in the fenugreek group compared to the control group. Another clinical trial was done for 20 days. Fasting blood glucose level decreased in the fenugreek group as well [116]. This result suggests that fenugreek can be used as diabetes treatment [116].

##### *Cinnamomum* (Cinnamon)

Not only has cinnamon been used as natural food preservative and spice, but it has been used in traditional medicine for treating rheumatism, wounds, diarrhea, headache, and colds [117]. In addition, cinnamon supplements are being used to treat asthma, arthritis, cancer, elevated cholesterol, T2DM, and various metabolic syndromes. Numerous studies have implicated cinnamon as diabetes treatment. Cinnamon may have functions similar to insulin [118].

An* in vitro* study with rat adipocytes suggests that cinnamon regulates insulin signaling [49]; this study proved that cinnamon amplifies the effect of insulin. Glucose oxidation by insulin and cinnamon was reduced as the concentration of wortmannin increased. Wortmannin is a fungal agent that inhibits phosphoinositide 3-kinase (PI3K) activity. This means that cinnamon has similar properties as insulin by activating PI3K in insulin signaling. Thus, cinnamon induces insulin signaling by activating PI3K. Furthermore, a protein tyrosine phosphatase (PTP-1) assay was done to show that cinnamon activates tyrosine phosphatase in insulin signaling. Research suggests that cinnamon regulates the insulin receptor, GLUT4 and tristetraprolin (TTP) expression in 3T3-L1 adipocytes [50]. In this study, two different forms of cinnamon were used: water extract and cinnamon polyphenol. Cinnamon polyphenol increased insulin receptor protein expression, but the water extract did not. Interestingly, when both water extract and cinnamon polyphenol were used in the treatment, GLUT4 protein expression increased. TTP is an inflammatory protein whose mRNA level was observed to be induced due to insulin treatment in a mouse fibroblast cell line. When both water extract and polyphenol were used, protein expression level and mRNA expression level of TTP were increased [49]. The results show that cinnamon can induce insulin receptor and GLUT4 protein expression. This indicates that cinnamon may reinforce the activity of insulin and could also have an analogous action as insulin. Therefore, cinnamon could have the potential to alleviate insulin resistance, which is one of many symptoms of obesity and T2DM.

##### *Dioscoreaceae* (Dioscorea)

Dioscorea is a tropical plant renowned for its anti-diabetic effects [71,119]. Insulin resistance was induced by tumor necrosis factor-α (TNF-α) to a FL83B mouse liver cell line [70]. Cells of a mouse liver cell line were treated with Dioscorea polysaccharide following insulin resistance induction. Glucose uptake increased when dioscorea polysaccharide was given. Furthermore GLUT2 protein expression, Akt, insulin receptor, insulin receptor substrate, and c-Jun *N*-terminal kinase (JNK) phosphorylation were increased in dioscorea polysaccharide treated subjects [70]. These results suggest that dioscorea polysaccharide improves insulin resistance and hyperglycemia. The mixture of Chinese medicinal herb was administered orally to fructose-rich diet fed Wistar rats [71]. This herb contained dioscorea among six different phytogenic compounds. Reduced blood glucose level was evident compared to control. Each phytogenic compound was extracted from the mixture and administered to fructose-rich diet fed rats. Blood glucose level was increased with mixture absent of dioscorea. Each phytogenic compound of the herb was administered individually to insulin resistant rats. Only rats receiving dioscorea showed reduction in blood glucose level and increase of plasma insulin level [71]. This indicates that dioscorea extract can reduce blood glucose by increasing plasma insulin level.

##### *Vaccinium* spp. (Blueberry)

Blueberry improves insulin resistance. Freeze-dried whole blueberry powder supplemented HFD was fed to mice for 8 weeks [26]. Results indicated that mice fed with blueberry had lower blood glucose level compared to the control group. Apparently, the accumulation of adipose tissue macrophage (ATM) is involved in the onset of insulin resistance [120]. M1 macrophage induces inflammation and causes damage to tissues [121]. M2 macrophage activates cell proliferation and tissue repair. Numbers of M1 and M2 ATM were reduced in mice fed a HFD-containing blueberry than mice fed HFD alone. In addition, ATM-associated inflammatory gene expression in epididymal adipose tissue, such as TNF-α and monocyte chemotactic protein-1 (MCP-1), were reduced in mice fed the blueberry containing HFD. Another study suggested that oral administration of blueberry with Labrasol can reduce blood glucose level [40]. In one study, blueberry was prepared by fermenting with *S. vaccinii* [41]. The fermented blueberry was administered to KKA^y^ mice, both acutely and chronically. Both acute and chronic administration groups showed reduction of blood glucose level compared to the control. PPAR has multiple functions, which includes metabolism of carbohydrate and lipid [42]. The blueberry fed Zucker rats displayed increased PPAR-α and PPAR-γ activity [42]. In addition, PPAR agonist drugs induce fat metabolism and ameliorate insulin resistance [42]. This may suggest that blueberry extract improves insulin resistance by activating PPAR [42]. Overall, blueberry extracts seem to reduce insulin resistance. The exact mechanism remains unknown, but results of numerous studies suggest that blueberry extracts improve insulin resistance in a manner that does not involve induction of insulin secretion [26,41,42].

##### *Glycyrrhiza* (Liquorice)

Amorfrutins extracted from *Glycyrrhiza* can be used to treat diabetes. Chemically synthesized amorfrutins was administered to HFD-induced obese mice [67]. Blood glucose, plasma insulin, and body weight were reduced when amorfrutins were provided. Plasma insulin level was further compared with low-fat diet (LFD)-fed mice. Plasma insulin level was higher in HFD-fed mice compared to LFD-fed mice. However, mice fed HFD containing amorfrutins had similar plasma insulin level to LFD fed mice [67]. Ethanol extract of *Glycyrrhiza* was administered orally to KKA^y^ mice [68]. Blood glucose and body weight was reduced, as was abdominal adipose tissue. Results from binding affinity assay indicated that *Glycyrrhiza* extract binds to PPAR-γ [67,68]. Out of many functions, PPAR-γ is involved in regulation of insulin sensitivity and glucose homeostasis [122,123]. This also suggests that extract of *Glycyrrhiza* can improve insulin sensitivity and hyperglycemia.

#### 3.2.2. Possible Therapeutic Compounds that Regulate β-cell Function

##### *Ervatamia microphylla* (Kerr)

Conophylline is a compound extracted from *Ervatamia microphylla*. It has anti-diabetic effects. Blood glucose level was reduced when conophylline was administered orally to streptozotocin (STZ)-induced diabetic rat [54]. Conophylline induced pancreatic β-cell proliferation [124]. A study on pancreatic stellate cells suggests that conophylline inhibits activation of pancreatic stellate cells [55]. Furthermore, an *in vivo* experiment with Goto-Kakizaki rats showed that conophylline reduced blood glucose level and increased plasma insulin level [55]. These studies indicate that conophylline reduces blood glucose level via inducing β-cell proliferation.

##### *Anoectochilus roxburghii* (Jewel Orchid)

Kinsenoside is a compound of *Anoectochilus roxburghii*, commonly known as jewel orchid. Oral administration of kinsenoside to STZ-induced hyperglycemic rats reduced blood glucose level [56]. Plasma insulin levels increased in the kinsenoside group due to enlarged pancreatic β-cells compared to control.

##### *Carica papaya* (Papaya)

Papaya is a tropical fruit available in numerous countries. This fruit has been used in traditional medicine for a long time. Extract of papaya decreased blood glucose level in STZ-induced diabetic mice [57]. According to histological analysis, the papaya extract administered group had enlarged pancreatic β-cells compared to diabetic mice indicating that papaya extract induces β-cell regeneration, thus increasing insulin synthesis and reducing blood glucose level [57].

##### *Silybum marianum* (Milk Thistle)

Silymarin is a mixture of several flavonoids extracted from *Silybum marianum*. Silymarin was administered orally to alloxan-induced diabetic rats [75]. These rats had lower blood glucose level compared to the control group. Alloxan destroys pancreatic β-cells by producing hydrogen peroxide and free radicals. Activities of pancreatic superoxide dismutase (SOD), glutathione peroxidase (GSHPx), and catalase (CAT) increased in the silymarin administered group more than in the control group [75]. Therefore, silymarin may have the potential to improve hyperglycemia by protecting pancreatic β-cells. Seed extract of silymarin was orally administered to 51 T2DM patients [76]. Participants were given 200 mg of silymarin or placebo tablets three times a day for 4 months. Fasting blood glucose level and glycosylated hemoglobin level were reduced in the silymarin-administered group compared to the control group. In another clinical trial, silymarin was given to diabetic patients who did not respond to glibenclamide [77]. Silymarin or placebo tablet was given as adjunct to glibenclamide to 59 T2DM patients. Fasting plasma glucose and glycosylated hemoglobin level were reduced in the silymarin group [77]. These studies suggest that silymarin is effective at ameliorating symptoms of T2DM.

##### *Nymphaea stellata* (Egyptian Lotus)

The common name for *Nymphaea stellata* is Egyptian lotus. Chloroform extract of *N. stellata* flower was orally administered to STZ-induced diabetic rats [72]. Blood glucose level began to reduce from day 7 of administration. Oral administration of *N. stellata* extract increased the plasma insulin level. Further investigation with immunohistochemistry indicates increase in pancreatic β-cells mass [72]. This indicates that extract of *N. stellata* could potentially regenerate pancreatic β-cells, which could be developed into a treatment for diabetes.

#### 3.2.3. Compounds with Multiple Anti-Diabetic Activities

##### *Capsicum* (Chili Pepper)

A few studies suggest that capsaicin has an anti-diabetic effect. When capsaicin was administered to Zucker diabetic fatty rats, a reduction of blood glucose level and higher plasma insulin levels was evident compared to the control group [62]. HFD-induced diabetic rats were fed a chili pepper powder supplemented diet [63]. Blood glucose level did not decrease in the chili pepper powder fed group. However, plasma insulin was higher in the chili pepper powder fed group than in the control group. The results suggest that capsaicin can reduce elevated blood glucose.

##### *Momordica charantia* (Bitter Melon)

Bitter melon has long been known to have an anti-diabetic effect. Aqueous extract of *M. charantia* was administered intraperitoneally to STZ-induced diabetic mice [58]. The *M. charantia* treated group showed reduced blood glucose level compared to the diabetic control group. A human clinical study was performed with 18 maturity-onset diabetes patients [59]. Juice of *M. charantia* or vehicle was given to the patients 30 min prior to measuring blood glucose level. Patients who took *M. charantia* juice showed lower blood glucose level compared to the vehicle control group. These results indicate that *M. charantia* is able to lower blood glucose in a diabetes mice model and in patients.

##### *Vitis vinifera* (Grape Vine)

Resveratrol is one of the polyphenols existing in *Vitis vinifera* extract [125]. Resveratrol treatment to muscle cell lines increased glucose uptake by activating AMPK [46,48]. Extract of *V. vinifera* inhibited the activity of glycogen phosphorylase b in a HepG2 cell line [47]. Glycogen phosphorylase b is an enzyme involved in the rate-limiting step of glycogenolysis, which converts glycogen into glucose-1-phosphate. Glycogen phosphorylase is a good target for treating T2DM since hepatic glucose level is increased in T2DM patients [123]. Thus suppressing the activity of glycogen phosphorylase can reduce hepatic glucose level in T2DM patients. Overall, extract of *V. vinifera* can reduce high blood and hepatic glucose levels in T2DM patients.

### 3.3. Possible Therapeutic Compounds for both Obesity and Diabetes

Obesity and diabetes have some common links and are connected closely to each other. The term “diabesity” was coined in the 1970s to emphasize the strong relationship between obesity and diabetes [126]. However, some current treatments for diabetes may lead to obesity. For example, sulfonylureas (insulin secretagogues) can induce weight gain as a side effect [69]. Effective treatments for both obesity and diabetes without side effects have not been discovered. Thus, we rely on phytogenic compounds to treat both obesity and diabetes due to fewer side effects than chemical pharmaceuticals [1]. Our review of some phytogenic compounds that have potential to treat either obesity or diabetes indicates at least the potential that some compounds may treat both diseases simultaneously in practice. In this section, phytogenic compounds that could potentially be developed into treatment for both obesity and diabetes are summarized.

*Vaccicum* spp. improves insulin resistance [26,100], suppresses appetite [43], and regulates lipid metabolism [45]. Anthocyanins extracted from blueberry have potential in the treatment of obesity and diabetes. Water extract of blueberry reduced food intake, body weight gain, body fat, and blood glucose level and activated PPARs [44]. In addition, *V.* *angustifolium* (wild blueberry) also contains anthocyanins that improve dyslipidemia by regulating genes related to lipid metabolism [45]. Anthocyanins might improve insulin resistance [40] and reduce body weight and regulate lipid metabolism.

*Capsicum* induces thermogenesis by activating BAT [65,127]. While the effect of capsaicin on energy expenditure remains unclear, it seems to improve hyperglycemia by increasing plasma insulin level [62]. As a result, blood glucose level is decreased. Thus, there is a possibility that capsaicin can induce thermogenesis and decrease blood glucose level simultaneously. However, as each study is performed in different models and using diverse conditions, further investigations are required to confirm the efficacy of *Capsicum*.

It is clear that extract of *Glycyrrhiza* affects both obesity and diabetes. This phytogenic compound can improve hyperglycemia [67]. *Glycyrrhiza* extract increased β-oxidation and decreased acetyl-CoA synthesis [69]. Clinical trials further suggest that *Glycyrrhiza* extract can reduce body fat [101,102,69]. Prior studies indicate that extract from *Glycyrrhiza* has the potential to attenuate symptoms of obesity and diabetes.

*M. charantia* extract can improve hyperglycemia [58,59] and reduce hyperlipidemia [61]. *M. charantia* extract can suppress adipocyte hypertrophy and reduce lipogenic gene expression, such as expression of FAS, ACC-1, and LPL [61]. In addition, *M. charantia* extract reduces SREBP-1c and resistin [105]. Thus, *M. charantia* may act as an inhibitor of lipogenesis and lipolysis stimulator. *M. charantia* extract could reduce hyperlipidemia and hyperglycemia.

*Cinnamomum* extract can lower hyperglycemia [49,50,99]. It is likely that cinnamon extract has a mimetic effect of insulin or amplifies the activity of insulin. *Cinnamomum* extract seems to reduce body fat composition [51,100]. Thus, it is possible that cinnamon extract could be used to treat both obesity and diabetes. However, the exact function of *Cinnamomum* and its relation to insulin [118], and the mechanism in reducing body fat composition remains unclear.

## 4. Conclusions

In this review, phytogenic compounds that affect obesity and diabetes have been discussed. While a simultaneous treatment for obesity and diabetes has been explored, no treatment is available. Obesity and diabetes shares some similarities that include inflammation and insulin resistance [128].

Due to unique characteristics of phytogenic compounds and similarities of obesity and diabetes, some phytogenic compounds may be used to develop treatment for both diseases. For example, extract of cinnamon can improve hyperglycemia and reduce body fat composition. Further investigations would provide support for the potential of cinnamon. Studies referred to in Section 3.3 were performed in different conditions and model systems. To verify the effect of the same compounds, identical experiments conducted with an animal model showing a phenotype of both obesity and diabetes, such as B6.Cg-*Lep*^ob^/J and B6.BSK(D)-*Lep*^db^/J mice, are needed. This mouse model has the physiology of both obesity and diabetes. Thus, it would be interesting to test if the phytogenic compounds mentioned in Section 3.3 could improve the phenotypes of obesity and diabetes. Although additional research is needed to confirm multiple aspects of the efficacy of phytogenic compounds, future studies on screening of phytogenic compounds adequate and effective for obesity and diabetes will contribute to the development of treatment options for these diseases.
